# "It's for a good cause, isn't it?" - Exploring views of South African TB research participants on sample storage and re-use

**DOI:** 10.1186/1472-6939-13-19

**Published:** 2012-07-25

**Authors:** Gerrit van Schalkwyk, Jantina de Vries, Keymanthri Moodley

**Affiliations:** 1 Centre for Medical Ethics and Law, Department of Medicine, Faculty of Health Sciences, Stellenbosch University, Stellenbosch, South Africa; 2 Institute for Infectious Disease and Molecular Medicine, Faculty of Health Sciences, University of Cape Town, Cape Town, South Africa

## Abstract

**Background:**

The banking of biological samples raises a number of ethical issues in relation to the storage, export and re-use of samples. Whilst there is a growing body of literature exploring participant perspectives in North America and Europe, hardly any studies have been reported in Africa. This is problematic in particular in light of the growing amount of research taking place in Africa, and with the rise of biobanking practices also on the African continent. In order to investigate the perspectives of African research participants, we conducted a study with research participants in a TB study in the Western Cape, South Africa.

**Methods:**

Semi-structured interviews were conducted using an interview guide which drew on the most prominent themes expressed in current literature on sample storage, re-use and exportation. Interviews were conducted in Afrikaans and subsequently translated into English by the same interviewer. Interviews were transcribed verbatim and analysed qualitatively.

**Results:**

The results of our study indicate that the majority of participants were supportive of giving one-time consent to the storage and re-use of their samples. The concept of research being for a “good cause” was a central prerequisite. Additionally, a significant minority requested that they be re-contacted if a future use was not stipulated on the original consent. There was also considerable variation in how participants understood the concept of a ‘good cause’, with participants describing three distinct categories of research, of which two were generally thought to constitute ‘good cause’ research. Research that was for-profit was considered to fall outside the spectrum of ‘good cause’ research. Participants displayed confidence in the abilities of the researchers to make future decisions regarding sample use, but seemed unaware of the role of ethics committees in either this process or more generally.

**Conclusions:**

Participants expressed a wide and complex range of views about issues of sample storage and re-use, and they showed a great deal of trust in researchers. Participants’ willingness to have their samples stored and re-used is consistent with findings from existing studies. However, in contrast to existing literature, participants were generally not in favour of for-profit research. Further research needs to be done to explore these ideas in other communities, both in South Africa and other countries.

## Background

Following the sequencing of the human genome, advances in genetics have opened up a plethora of research strategies to investigate disease [1]. Pivotal to such strategies is the long-term storage of samples, either as isolated collections or in centralised biobanks, and the re-use of samples and data for multiple projects [2].

A number of ethical challenges have been identified in relation to the long-term storage and secondary use of samples and data. In particular, ethical questions about the governance of such collections and the compatibility of secondary use with informed consent have been identified and discussed [3,4]. Some authors have argued in favour of a process where participants sign consent on one occasion, thereby authorising the researchers to use their sample for several subsequent projects. There are different viewpoints about whether or not such broad consent is sufficiently informed [4,5]. Those in favour have argued that the purpose of informed consent is chiefly to respect the autonomy of the patient, and that broad consent does not compromise this aim [4]. On the contrary, others have argued that broad consent subverts the principle of good informed consent by providing little to no information about ‘future use’ [5].

In addition to consent, another prominent concern relates to the concept of sample ownership. Specifically, it is unclear to what extent participants should be and are able to retain rights to their donated samples [6]. The issue becomes clouded even further when future usage includes research that has the potential to generate profit.

Moving beyond the theoretical debate, a number of studies have been conducted that examined the views of research participants regarding the acceptability of long-term sample storage and consent. In an extensive review of the topic, Wendler found more than 80 % of respondents in the vast majority of studies reviewed reported that they would consent to donating a sample for use in a biobank [7]. Additionally, in the six studies included in the review that addressed the issue of informed consent, between 79 – 95 % of participants were in favour of one-time, general consent, and were happy to rely on ethics committees to make further decisions regarding use.

It would therefore appear that there is broad public support for research involving biobanks, but important challenges remain [8,9]. For instance, what remains unclear is how to best obtain informed consent for long-term sample re-use when future uses are not currently known [10,11]. Furthermore, a drawback of this literature is that the majority of research investigating participants’ perspectives has focussed on populations in the US and Europe. Data on the views of African research participants remains scarce despite an increase in the number of research projects taking place on the continent [12,13]. Southern Africa is regarded as fertile ground for a wide range of research endeavours due to the enormous burden of infectious disease in the region, highly skilled medical researchers and large numbers of treatment naïve patients [14]. South Africa is positioned at the epicenter of the HIV pandemic, and HIV research as well as HIV biobanking is flourishing scientifically. There are currently 1390 clinical trials registered on the South African Clinical Trials Register hosted by the Department of Health. Two years ago (2010) there were 946 trials registered [15]. This indicates a significant growth of clinical trial research over the past 2 years.

We are aware of only two reported research studies that investigate the perspectives of African research participants regarding sample storage and re-use, in Egypt [16] and in Uganda [17]. The Ugandan study (n = 343) found that a large majority (95 %) of participants would consent to their sample being re-used without additional consent, subject to approval by an Institutional Review Board (IRB). On the other hand, the study in Egypt (n = 600) found that just under half (44.3 %) of the participants felt that consent forms should include a separate section relating to storage and future use of samples and data. Both studies used quantitative sociological methods to invite participant viewpoints. To our knowledge, no qualitative research studies have been reported in Africa, including Southern Africa. Considering the contextual complexity of these issues, and the vast cultural and geographical diversity on the African continent, we believe it is of paramount importance to seek the views of African research participants on these issues. Our study aimed to provide a preliminary exploration into these issues, specifically seeking to qualitatively explore the views of research participants on sample storage and re-use.

## Methods

In order to explore the views of South African research participants on sample storage and re-use, we set out to conduct a qualitative sociological research study with participants in a current tuberculosis (TB) research project in a high density, low-income urban area in the Western Cape, South Africa. The aim of the TB study was to detect potential biomarkers of protective immunity in a high risk TB population. The TB study involved the collection of blood and saliva samples on three occasions over 2 years. The TB study participants were healthy and close contacts of people who had been diagnosed with TB, and had all consented to donate samples that would be stored in a biobank with the potential for future use. By the time that we conducted our research, the TB study site had enrolled approximately 500 participants. For our study, we approached participants in the TB study, and conducted semi-structured interviews with 20 individuals. Participants were selected at random with the assistance of the nurse who was acting as the site co-ordinator. The nurse provided us with a list of participants who were still active in the study, and who were due to have a follow-up visit in the near future. Recruitment was done both by contacting participants telephonically, or by requesting their involvement during their follow-up visits.

The aim of the interviews was to collect participants’ viewpoints on issues of sample storage and re-use and to examine the acceptability of these. In addition, viewpoints regarding how informed consent for these practices could be optimised were explored. The initial interview guide was developed by the research team, and discussed with an investigator of the TB project and with one of the project nurses. The interview guide was tested with two sixth year medical students to ensure that questions were clear and that they followed a logical order. See Table 1 for a summary of the interview guide.

**Table 1:** Summary of themes explored in interviews

**Sample storage**
- Duration
- Method of storage
- Different sample types
**Sample ownership**
- Donation
- Access
**Re-use of samples**
- General thoughts
- Types of research
- Goals of research
- Commercial research
**Informed consent**
- Preferred method
- Opinion about “one-time” consent
- Role of ethics committee
- Role of community leaders

Interviews were conducted in Afrikaans by the same interviewer. The interviews were semi-structured, consisting of between three and five questions related to each theme, with specific prompts that were used as needed. A total of 20 interviews were conducted, by which stage preliminary analysis of the data suggested that no new major themes were emerging. After formal analysis of the data this view was confirmed, and no further interviews were conducted.

Interviews were recorded and transcribed verbatim. Coding was done chiefly by one researcher, in consultation with the other two researchers on the team. Initial coding of the data generated themes, which were included in a second round of coding [18]. All three members of the research team discussed the coding of the data. Relationships were established between themes and hierarchies established based on input from all three investigators. Analysis was done using QRS NVivo version 9 [19]. Transcripts were coded in the language in which they had been conducted, where after the quotes that were selected for inclusion in this manuscript were translated verbatim into English.

Approval was obtained from the Stellenbosch University Health Research Ethics Committee (Ref N11/04/118). Informed consent was obtained from all participants.

## Results

Sixteen of the participants were female and 4 were male which broadly reflects the gender bias that was present in the TB study itself. All of the participants were Afrikaans-speaking and of mixed ancestry. As such, this study likely informs more on the views of people with mixed ancestry than on any other segment of the population in our study area. The average age of the participants was 34 years, ranging from 18 to 65. The mean number of years of schooling was 10.5. This ranged from participants who had only completed 4 years of schooling to participants who had received tertiary education. The mode was 9 years of schooling, which is consistent with the minimum legal requirement for schooling in South Africa. Fifteen of the 20 participants were unemployed.

### Issues regarding storage

Participants were asked how they felt about the possibility that their specimens could be stored after the initial round of tests had been conducted. Research participants generally seemed to be unconcerned about sample storage. The most commonly cited reason given was that once the sample had been donated, they would have no use for it, and storage would not affect them.

"Yes I accept that (the blood being stored), what am I going to do with it, I can do nothing with that blood, I can’t put it back in."

And,

"It’s blood that’s already been drawn, so it won’t be an issue for me…. It’s not something you can put back."

But most participants (17 of 20) indicated that they would only consent to storage if samples would be stored securely and only be accessible to the research staff.

### Sample ownership

Some divergence of opinion arose when asking participants whether they viewed the sample as still being their property. Two participants felt that the sample remained theirs, and that they reserved the right to either request for it to be returned, or have it destroyed. The remainder of the participants felt that the sample became the property of the researchers.

"No, I don’t want to have access anymore. It’s not mine anymore. They are using it now."

However, although the majority of participants felt that the sample was no longer their property, it was clear that they did not feel that surrendering ownership meant that they lost all their rights. For instance, some participants insisted that if revenue were ever to be made from research using their sample, they would want to be re-contacted to discuss how they or the community would benefit.

"The thing is I gave it to them specifically to experiment with. I didn’t give it to them to make a profit."

### Re-use and the “good cause”

Next, we asked participants for their views on sample re-use. When asked about the possibility that their donated samples could be re-used, all participants indicated that they would consent to this. But many participants (19 out of 20) described some conditions for re-use. Most notably, participants felt that the purpose of sample re-use should be to support research that would constitute a “good cause”.

"Look, as long as it’s for a good cause (re-use), if it will, how can I say, enrich people’s lives, then its okay."

The main condition for re-use to be for a ‘good cause’ seemed to be based on altruistic motivations.

"Yes, they use it to the benefit of others. It’s not only for me, because a person must not always think about yourself. You must think maybe this can affect other people also…"

For 17 out of 20 research participants, altruism was the main motivation to agree to participation in the TB research study. Similarly, it was most commonly given as a motivation for accepting sample re-use.

Considering the prominence of ideas about a good cause as a requirement for sample re-use, we spent considerable time exploring what participants would consider a good cause. In the course of this exploration, three distinct categories of use emerged.

#### Community benefit

The first category is where research was conducted that could have a direct benefit for the community. The most commonly cited example was that of research on tuberculosis, which is highly prevalent in the community.

"It’s actually to the benefit of others, because maybe in your blood there is something specific they can see in other people, and then they can prevent that this (TB infection) happens again."

There were also examples where community benefit was understood in a broader sense.

"I mean, you are doing something […] I see it this way, if I can help with any medical research and it’s a bit of blood, it not litres and litres, its fine with me"

And

"Like sometimes, it is maybe for cancer and things like that, it’s for good purposes that they are going to use it, and not for something else"

One participant, however, was more sceptical about the likelihood that any benefit would flow from research to participants or their communities.

"You can say, yes, this is for the community with the TB problem, they will agree but at the end of the day, all is said and done, they were pricked with needles by people in white coats and they got nothing out of it"

The same participant went on to describe what he would consider to be an appropriate form of benefit.

"But if they can get a small something out of it, you know? Even if it’s just a letter to say thank you, the study was a success, you know? A box of biscuits maybe? That kind of thing."

This point of view was in contrast with that of most other participants, who emphasised their positive view of TB research as being a source of community benefit.

#### Academic and institutional benefit

The second category of research for the good cause was research that could be regarded as more academic in nature – thus not something that would be of obvious direct benefit to the local community. Nine out of 20 participants would support this kind of re-use. There appeared to be a sense amongst these participants that if they could contribute to scientific research, even of a more abstract nature, then they would be happy to do so.

"Yes I will say that is okay. Why, because, he can go ahead with his career (the researcher), and he wants to progress, then I am not concerned."

In some instances, while participants expressed support for sample re-use for research of a more academic nature, they also expressed the hope that such research could be beneficial to themselves or the community in the future. For instance, one participant suggested that by contributing to academic research, the researcher would eventually be in a position to cure a disease like a HIV. Another participant hoped that by supporting the university, there was an increased chance that they would be able to offer scholarships to members of the community.

#### Research for commercial purposes

A third category of use, and one that respondents did not consider to be an example of re-use for a good cause, was research done for the purposes of making a profit. All of the participants expressed concern with this idea, and would not wish for their sample to be re-used for such a purpose.

"Respondent: If it is for the institution and entirely for research […] maybe they can buy new equipment or whatever they need in the lab […] then the number of people I am helping multiplies, because then the research can carry on, yes."

"Interviewer: [… And] if it is something that could lead to profit for a corporation?"

"Respondent: No, that is something completely different. That won’t work at all."

Participants furthermore indicated scepticism about whether or not they would be informed if any revenue was made following the use of their samples.

"In any case they aren’t going to tell me they are going to do it. It is going to happen in secret. So, who is going to tell me they have done this and invested in that. It’s not as if they are going to come tell me."

Some participants considered that they would consent if they were to receive a share of any profits. Other participants again felt that profit could be justified if there was some form of community re-investment. However, it must be emphasised that this idea was largely foreign to many of the participants, and it was at times difficult to establish clear boundaries for when research was actually considered to be “for profit”. Therefore, our data must be seen as pointing towards a focus for further investigation before conclusions can be drawn.

### Re-use and keeping participants informed

In addition to constructing an in-depth understanding of the contexts in which participants would regard re-use to be acceptable, the interviews also sought to examine what respondents considered to be the most appropriate way to obtain and maintain informed consent for secondary re-use and sample storage. All participants favoured a one-time consent process, although two broad sets of perspectives emerged when it came to the details of how far this process could go. Some participants (5 out of 20), felt that all intended usage should be specified at the outset, and that consent should be renewed if a previously undescribed application was being considered.

"Yes, it’s still my blood and they must come ask me if they want to do something else with it."

And,

"Because, I mean, you can’t make a list of four things, and then I sign and then you add another thing, do you understand?"

The second perspective was that of participants who would be happy for their samples to be re-used for *any* research that met the essential requirement of being for a “good cause”. The reasons for this were varied – some participants felt that sample re-use would make no difference to them, given that the sample had already been collected. They seemed also concerned that they might be unnecessarily troubled if they had to re-consent for previously undefined uses.

"No, I wouldn’t like to be re-contacted every time a test is done to come sign a form, because I have already donated the blood."

Other participants indicated to rely on the researchers’ specialist knowledge to decide on the appropriateness and necessity of sample re-use.

"Interviewer: And do you trust these people to decide what is a “good cause”? You don’t feel they should come to you and ask whether you feel it is a good cause?"

"Respondent: No, I think it would depend on them. They are more in the, it’s more in their field. They know what to do."

And,

"Yes, if it has to do with the community then it would be very good (to have samples re-used), and if they (the researchers) know it’s a good choice they are making on my behalf then it will be fine with me."

Participants generally seemed to trust researchers to act in their best interests, and they considered them to have good intentions.

"Yes, and the reason is (for trusting) they do it with a good heart. Look, they take the time to try and find out where they can find solutions for TB and such things"

However, as illustrated earlier, there were instances where participants felt that researchers would not always act in their best interests, and trust was therefore not unanimous amongst participants. Although attempts were made to explore the issue of trust in ethics committees, none of the participants were aware of what an ethics committee was, or indeed, that the research in which they were participating had been subjected to ethical review. This greatly limits the reliability of their responses to this line of enquiry. Further research will be required to understand how to balance these observations in the consent process in medical research in South Africa.

Interestingly, 6 participants indicated that they would like to be kept informed of sample use, even if they did not require renewal of their consent.

"They don’t need to get permission again, but I also don’t want to be kept in the dark. I would appreciate being kept up to date on what it is being used for, or maybe they need more. Yes, I would like to know."

## Discussion

Our study uncovered a wide range of views regarding sample storage, re-use and consent required for these two components of the research process. In general, participants in our study seemed to be supportive of sample storage and re-use, which is consistent with existing literature [4]. However, they did indicate that certain types of re-use would be more acceptable than others. Of particular interest is that our participants appeared to have little to say about the *nature* of the research. This is in contrast to the survey by Abou-Zeid et al. [17], where there was a significant difference between the number of participants who would consent to research involving a blood sample and those who would consent to genetic research. Our participants appeared to have limited understanding of what genetic research was, making it impossible to reliably assess how they would feel about such applications.

Participants were, however, able to differentiate research practises based on their aims. We categorised the examples given by research participants into three categories. These are: re-use for research that has a direct health benefit for the community; research that would be of benefit to the institution or field in a more general sense; and ‘for profit’ research. The first two fall under what one participant called ‘research for a good cause’, of which participants were generally supportive. Research in the third category, ‘for profit’, was not generally supported by research participants. This is an interesting finding, particularly in the context of existing studies. Abou-Zeid et al. reported that only 32.8 % of participants in their study would desire a share in any commercial profits [16]. Although our question was slightly different, it does suggest that the participants in our sample had more concerns with for profit research than has been reported in previous studies.

Our data suggests that participants were generally in favour of a one-time consent procedure when donating samples for storage and re-use, although a minority felt that re-consent should be sought if the re-use was for a purpose not initially specified. This is in slight contrast to the findings of Wendler et al. in Uganda, who reported that 95 % of participants would be happy not to be re-contacted, as long as the re-use was approved by an Institutional Review Board [17].

Participants based their preference for a one-time consent on the perceived expertise of the researchers, and on their expectation that researchers would act in their best interest. Participants furthermore indicated that they would not really be affected if samples were used multiple times, once the sample had been taken, and that it may be burdensome to be re-contacted for subsequent uses. When expressing confidence in the researchers’ good intentions, this seemed to relate more to the personal characteristics of the researcher than to his or her institution. This finding would suggest that relying on ethics committees to make re-use decisions can be problematic in a community where the focus of participants’ trusts is more specifically on the actual researchers. Comparing this finding to existing research is problematic, since it is primarily quantitative in nature. For example, although the study in Uganda found that participants were in favour of institutional review boards making further research decisions on their behalf, we cannot be sure that participants fully understood the concept of what an institutional review board was. In the previously mentioned review by Wendler [7], the comment is made that “Data from more than 33000 people around the world support offering individuals a simple choice of whether or not their samples can be used for research purposes, with the stipulation that an ethics committee will decide the studies for which their samples are used”. We would argue that if such an approach is to be employed, further research would be needed to investigate participants’ understanding of research ethics review as a component of the research process.

Participants indicated that they would like to be informed about the research they are involved with, in a way that was distinct from a second informed consent procedure. This desire to be kept in formed is consistent with findings from several other studies [7]. More research needs to be done to assess the feasibility and possible impact of these types of feedback in the South African context, which on initial exploration appear to have the potential to increase both participant trust and satisfaction. This is particularly relevant in the case of informing participants about the eventual benefits of any research project, which can serve both to address deficiencies of trust and misunderstandings about research outcomes.

The primary limitation of our study is the fact that participants appeared to be uninformed about several aspects of the research process. In particular, this limited our ability to explore two significant themes pertaining to biobanking practises – specifically, sample exportation, and the use of samples for specific research types (such as genetic research). Gaining well considered input on these issues remains a important challenge for the future. An additional limitation of our study is that we report on a relatively specific subgroup. Further research will be required to assess the degree to which the concerns we identified are of relevance in the broader population.

## Conclusions

In conclusion, the results of our study serve to highlight that fact that participants display a wide and complex range of views regarding these issues. Broadly, participants appear to be in favour of having samples stored and re-used for research that is for a “good-cause”, a category to which for-profit research appeared to be the only significant exclusion. How participants understood “for-profit” was not clear, and represents an important avenue for further research. Further research also needs to be conducted in order to explore these ideas in different communities, both in South Africa and other countries, so as to improve our ability to align research practices with the preferences of the population under study.

## Competing interests

The authors declare that they have no competing interests.

## Authors’ contributions

GvS designed the interview guide, conducted the literature review, identified a research site and collected and analysed the empirical data. He also prepared a first draft of this paper. JdV assisted in literature review and study design, and in analysis of project data. KM conceptualized the research project, conducted the literature review and supervised and co-ordinated data collection. KM and JdV commented on drafts of the manuscript. All authors read and approved the final manuscript.

## Pre-publication history

The pre-publication history for this paper can be accessed here:

http://www.biomedcentral.com/1472-6939/13/19/prepub
